# Exposure to phthalates aggravates pulmonary function and airway inflammation in asthmatic children

**DOI:** 10.1371/journal.pone.0208553

**Published:** 2018-12-17

**Authors:** Young-Min Kim, Jihyun Kim, Hae-Kwan Cheong, Byoung-Hak Jeon, Kangmo Ahn

**Affiliations:** 1 Environmental Health Center for Atopic Diseases, Samsung Medical Center, Seoul, Korea; 2 Department of Pediatrics, Samsung Medical Center, Sungkyunkwan University School of Medicine, Seoul, Korea; 3 Department of Social and Preventive Medicine, Sungkyunkwan University School of Medicine, Suwon, Korea; Stony Brook University, Graduate Program in Public Health, UNITED STATES

## Abstract

**Introduction:**

Studies on the associations between phthalate exposures and respiratory outcomes are limited. We investigated the association of phthalates exposure with pulmonary function and airway inflammation in asthmatic children.

**Methods:**

Fifty-six children with asthma living in Seoul Metropolitan Area, Korea aged 6–16 years were enrolled. Their pulmonary function including forced expiratory volume in 1 sec (FEV_1_) and peak expiratory flow rate (PEFR) were measured, and the fractional exhaled nitric oxide (FeNO) as a marker of airway inflammation was examined repeatedly up to four times during the study period. Urinary levels of mono-(2-ethyl-5-hydroxyhexyl) phthalate (MEHHP) and mono-(2-ethyl-5-oxohexyl) phthalate (MEOHP), metabolites for di-(2-ethylhexyl) phthalate (DEHP), and mono-n-butyl phthalate (MnBP), a metabolite of di-n-butyl phthalate (DnBP), were also measured on the same days. The effects of phthalate metabolites on the respiratory symptoms were analyzed using linear mixed effect models with adjustment for potential cofounders.

**Results:**

An increase in phthalate metabolites was associated with a decrease in pulmonary function and an increase in FeNO in asthmatic children. As one natural log-unit (ln-unit) levels of urinary MEHHP and MEOHP increased, FeNO levels on the same day increased by 19.47 ppb [95% confidence interval (CI): 9.28, 29.67] and 17.93 ppb (95% CI: 5.86, 30.01), respectively. An increases in the urinary level of MEHHP, MEOHP, and MnBP by one ln-unit was associated with a decrease in PEFR on the next day by 12.17 L/min (95% CI: 2.59, 21.74), 10.80 L/min (95% CI: 0.29, 21.32), and 13.65 L/min (95% CI: 5.07, 22.24), respectively.

**Conclusion:**

Phthalates, especially DEHP, may worsen pulmonary function and airway inflammation in asthmatic children. To control asthma symptoms, exposure to phthalates needs to be avoided.

## Introduction

Chemicals emitted from environmental materials and personal care products have a variety of adverse effects on animal and human health [1–3]. Among environmental chemicals, phthalates have been considered as risk factors for asthma and allergic diseases [4–7]. Phthalates, a group of high-production volume compounds added to plastics to confer flexibility (e.g., plasticizers in polyvinylchloride plastics), are widely used in personal care products, medications, and food processing and packaging materials [8–10].

The ubiquitous presence of phthalates and the potential consequences of human exposure have raised concerns, particularly in children, because they are more likely to be exposed to phthalate than adults through ingestion of dietary sources and sucking dust from phthalate-containing products [11, 12]. Recently, phthalates commonly found in households including vinyl products, toys and electronics have been reported to influence respiratory and asthma symptoms [5–7,13–15]. For example, according to a study in 3 to 9 years old children [6], mono-n-butyl phthalate (MnBP), a metabolite of di-n-butyl phthalate (DnBP), was related to diagnosed asthma and daytime respiratory symptoms. Bekö et al. [13] demonstrated that di-(2-ethylhexyl) phthalate (DEHP) metabolites were associated with the higher risk of wheeze and bronchitis. In a study of 244 children, increases in fractional exhaled nitric oxide (FeNO), a marker of airway inflammation, were associated with log-unit increases in urinary concentrations of metabolites of diethyl phthalate (DEP) and butylbenzyl phthalate (BBzP), but not with concentrations of DEHP or DnBP metabolites [14]. In a Spanish birth cohort study by Gascon et al. [16], the maternal urinary DEHP and mono-benzyl phthalate (MBzP) levels during pregnancy were significantly associated with asthma at age 7 years, suggesting that high-molecular weight phthalates increase the risk of asthma development in childhood. In the Taiwan Birth Panel Study, phthalate exposure at age 9 years was negatively associated with forced expiratory volume in 1 second (FEV_1_) and forced vital capacity (FVC) [17]. However, the adverse effect of phthalate on the airway is still controversial, because Vernet et al. [15] found no clear deleterious associations between in utero exposure to phthalates and respiratory outcomes, including wheezing until age 5 years, and bronchiolitis/bronchitis episodes until age 3 years in offspring. Further studies to show the association of phthalates exposure with aggravation of asthma using objective measurements are needed.

We, therefore, aimed to investigate whether exposure to phthalates exacerbates pulmonary function and airway inflammation in asthmatic children.

## Methods

### Study participants

In the present study, 56 asthmatic children (35 boys and 21 girls) aged 6–16 years living in the Seoul Metropolitan Area in Korea were enrolled as a panel and followed from October 2013 to February 2015. Asthma was diagnosed by physicians based on typical clinical symptoms in the last 12 months and positive airway hyper-responsiveness. Asthma symptoms include recurrent wheezing, cough or breathing difficulties. Airway hyper-responsiveness was confirmed by either provocation concentration of methacholine causing a 20% fall in FEV_1_ (PC_20_) less than 8 mg/mL or bronchodilator responses of at least 12% in FEV_1_.

Skin prick tests were performed to detect specific IgE against common inhalant allergens including *Dermatophagoides pteronyssinus*, *D*. *farinae*, tree pollen mixture, grass pollen mixture, weed pollen mixture, cockroach, cat, dog, *Alternaria alternata*, and *Aspergillus fumigatus* (Allergopharma, Germany). Histamine was used as a positive control and normal saline was used as a negative control. A positive response to the skin prick test was determined when wheal size was ≥ 3 mm and controls showed adequate reactions.

Written informed consent was obtained from parents or guardians of all participating children. The study protocols were reviewed and approved by the Institutional Review Board (IRB) at Samsung Medical Center (Approval number: 2013-05-009).

### Exposure to phthalates

To assess the exposure to phthalates, we measured the urinary concentrations of phthalate metabolites including mono-(2-ethyl-5-oxohexyl) phthalate (MEOHP) and mono-(2-ethyl-5-hydroxyhexyl) phthalate (MEHHP), biomarkers of DEHP, and MnBP, a biomarker of DnBP. We selected the biomarkers for DEHP and DnBP to assess phthalate exposure because the concentrations of urinary DEHP and DBP metabolites in Korea are relatively higher than those reported in US and other countries [18]. In addition, the concentrations of DEHP metabolites were higher among children compared to those in the older age group [19]. The first urine in the morning and the last urine before sleep at night were collected from each child up to six times on different days.

Urine samples were collected in sterile cups, stored in -80°C freezer up to 3 months before analysis. Concentrations of the three phthalate metabolites (MEOHP, MEHHP, and MnBP) were analyzed using a high performance liquid chromatography-mass selective detector (HPLC-MS/MS, Agilent 1200 series, Santa Clara, CA, USA). Method detection limits (MDLs) of MEHHP, MEOHP and MnBP were 0.186 μg/L, 0.260 μg/L, and 0.423 μg/L, respectively. The concentrations of the phthalate metabolites were adjusted with creatinine concentrations.

Based on the levels of urinary phthalates metabolites, we estimated the daily intake (DI) of phthalates in asthmatic children by adopting physiologically based pharmacokinetic modelling (PBPK) to compare with reference doses (RfD) recommended in U.S. Environmental Protection Agency (USEPA) [20]. We used urinary metabolite excretion factors that have been established in metabolic studies before [20,21] and extrapolated the amount of ingested parent phthalate. The formula used to estimate the DI of phthalates is as follows:

$$DI=\frac{UE\times CE}{Fue\times 1000}\times \frac{{MW}_{d}}{{MW}_{m}}$$ (1)

Where, *DI* denotes daily intake (μg/kg-day), *UE* is the urinary concentration of metabolite per gram creatinine (μg/g), *CE* is the creatinine excretion rate normalized by body weight; *Fue* is the creatinine excretion to total elimination (*k*_*u*_*/k*_*total*_)_,_ and *MW*_*d*_ and *MW*_*m*_ represent the molecular weights of the diesters and metabolite, respectively. Parameters for *Fue* were 0.69 for MnBP [22] and for MEHHP and MEOHP 0.23 and 0.15, respectively [23].

### Respiratory outcomes

For the evaluation of pulmonary function, the measurements of FEV_1_, the percentages of FEV_1_ to FVC (FEV_1_/FVC(%)) and forced expiratory flow at 25–75% (FEF_25-75_(%)) were conducted using Vmax encore (Cardinal health, USA) on the same day as urine sampling. These spirometric tests were performed up to four times on different days when each subject did not manifest common cold. Rescue medicine such as bronchodilators was not used on the measurement day. Trained technicians assisted the children in performing pulmonary-function maneuvers in accordance with the standards of 2005 European Respiratory Society/American Thoracic Society (ERS/ATS) recommendations. The maximum values among 3 acceptable measures recommended by the ATS/ERS were used for the analysis of spirometric data for each subject. The airway obstruction was assessed by measuring the peak expiratory flow rate (PEFR) in the morning by patients or guardians using Mini-Wright (Clement Clarke International Ltd., Harlow, UK) on the same day as the spirometric tests. Airway inflammation was evaluated by measuring FeNO using Niox mino (Aerocrine, Solna, Sweden). For the analyses of the relationship between phthalate biomarkers in urine and respiratory outcomes, we measured the metabolites of phthalates in the first urine in the morning when analyzing FEV_1_, FEV_1_/FVC(%), FEF_25-75_(%), and FeNO measured in the morning at hospital. Morning PEFRs were matched with the first urine in the morning, while the evening PEFRs were with the urine collected at night.

### Statistical analyses

Natural log (ln)-transformed phthalate metabolites were used because their distribution was skewed and not normally distributed according to the Shapiro-Wilk W-test.

Two to eight observations with phthalate metabolites and respiratory outcomes were made in individual children. Considering the repeated measurement of the variables, a linear mixed effect (LME) model was used to estimate the association between the ln-transformed phthalate metabolites and respiratory outcomes. Age, sex, body mass index (BMI), urinary cotinine level, and the use of controller medication were adjusted in the model as they are related to pulmonary function. The basic model specifications are as follows:

$$E(Y)=\beta_{0}+\beta_{1}*ln\left(PM\right)+\sum CF_{i}+\gamma (subject)+e$$ (2)

where *E(Y)* is the expected expression of pulmonary function or inflammation; PM refers to natural log-transformed levels of phthalate metabolites, and *CF*_*i*_ indicates the confounding factors including age, sex, BMI, urinary cotinine level, and the use of controller medication; *γ* denotes the random effect for each subject.

The pulmonary function is significantly associated with outdoor temperature [24] and air pollutants such as particulate matter with an aerodynamic diameter of 10 μm or less (PM_10_) [25]. We, therefore, included ambient PM_10_, outdoor temperature, and relative humidity (RH) as covariates in the LME models and compared the results from the adjusted models with those from the basic model, Formula 2. Data for PM_10_ were obtained from the National Institute of Environmental Research, while temperature and RH values were collected from the Korean Meteorological Administration. We used PM_10_ level on the previous day as a confounding factor when we fitted models for lung functions (morning PEFR, FEV_1_, FEV_1_/FVC(%), FEF_25-75_(%)) and FeNO measured in the morning. In contrast, we used PM_10_ concentrations on the same day for analyzing evening PEFR. Daily 24 hour-average values of temperature and RH were used on the same day as the measurements of respiratory outcomes and urinary biomarkers. Delayed effects of phthalates exposure on PEFR were also analyzed up to 2 days.

We also used penalized regression curves of a generalized additive mixed model (GAMM) to examine the relationship between phthalate biomarkers in urine and respiratory outcomes. All the predicting variables were included in the GAMM model adopting smoothing splines with adjustment for age, sex, BMI, ambient PM_10_, outdoor temperature and RH, urinary cotinine level, and controller medication.

All the procedures were conducted using R version 3.2.2 (The Comprehensive R Archive Network: http://cran.r-project.org) using the “mgcv” package (version1.8–7) for GAMM and “lme4” package (version 3.2.3) for LME model fitting. All tests were two-sided, and an alpha level of less than 0.05 was considered significant.

## Results

We enrolled 56 asthmatic children (35 boys and 21 girls) in this study. Table 1 shows the characteristics of the study population. The average age and BMI was 8.6 years (SD = 2.5) and 17.5 kg/m^2^ (SD = 2.7), respectively. Controller medications such as leukotriene modifier or inhaled corticosteroid were used in approximately 88.8% of the patients. There were no differences in demographic characteristics between boys and girls.

**Table 1:** Characteristics of the study subjects.

Variable	Total (N = 56)	Boys (N = 35)	Girls (N = 21)	*P*-value^a^
**Age (year)**	8.6 ± 2.5	8.5 ± 2.6	8.7 ± 2.4	0.793
**Height (cm)**	132.4 ± 16.3	133.9 ± 17.7	130.0 ± 13.6	0.374
**Weight (kg)**	31.7 ± 11.6	32.6 ± 12.8	30.2 ± 9.2	0.420
**BMI (kg/m^2^)**	17.5 ± 2.7	17.5 ± 2.9	17.5 ± 2.5	0.997
**Total IgE (U/L)**	683.8 ± 545.4	707.2 ± 556.1	646.5 ± 540.1	0.699
**PC_20_ (mg/mL)**	2.4 ± 1.7	2.5 ± 1.7	2.4 ± 1.6	0.804
**Sensitization to inhalant allergens^b)^ (%)**	88.4	92.3	82.4	0.256
**Use of controller medication (%)**	88.8	85.6	90.9	0.301

Data are expressed as mean ± standard deviation

^a^Test for differences between boys and girls: chi-squared test for sensitization to inhalant allergens and the use of controller medication, and *t*-test for means of remaining variables

^b^Sensitized to house dust mites (*Dermatophagoides pteronyssinus*, *D*. *farinae*), pollen mixtures (tree, weed, grass), cat, dog, cockroach, *Alternaria alternata* or *Aspergillus fumigatus*. BMI, body mass index; PC_20_, provocation concentration of methacholine causing a 20% fall in forced expiratory volume in one second (FEV_1_).

A total of 156 urinary samples were collected from 56 children. The urinary levels of phthalate metabolites in asthmatic children are shown in Table 2. Geometric means of MEHHP, MEOHP, and MnBP were 49.6 μg/g-creatinine [interquartile range (IQR), 35.2–85.7], 38.4 μg/g-creatinine (IQR, 27.2–60.6), and 71.8 μg/g-creatinine (IQR, 45.8–100.5), respectively. No significant differences were found in the levels of phthalate metabolites between boys and girls, although the levels in boys appeared to be higher than in girls. Outdoor environmental indicators including PM_10_, temperature, and RH on the same days as the urinary metabolites measurements are also shown in Table 3.

**Table 2:** Distribution of urinary metabolites and outdoor environments.

		N	Total (N = 56)	Boys (N = 35)	Girls (N = 21)	*P*-value^a^
**Urinary phthalate metabolites** (μg/g-creatinine)	MEHHP	156	49.6 (35.2–85.7)	51.1 (36.0–89.8)	47.6 (34.0–65.8)	0.297
	MEOHP	156	38.4 (27.2–60.6)	38.6 (28.1–64.8)	38.2 (26.1–55.7)	0.426
	MnBP	156	71.8 (45.8–100.5)	76.3 (46.2–119.6)	66.0 (45.7–84.5)	0.123
**Urinary cotinine** (μg/g-creatinine)		156	0.9 (0.2–3.1)	0.8 (0.1–2.5)	1.0 (0.2–3.6)	0.168
**PM_10_ (μg/m^3^)**		154	47.8 ± 29.6	46.0 ± 27.0	50.3 ± 32.8	0.481
**Temperature (°C)**		156	10.1 ± 8.4	10.0 ± 7.9	10.2 ± 9.1	0.905
**Relative humidity (%)**		156	61.9 ± 14.0	61.8 ± 14.1	62.0 ± 13.9	0.926

Data are expressed as geometric mean and interquartile range for urinary phthalate metabolites and cotinine levels, and also expressed as mean ± standard deviation for PM_10_, temperature, and relative humidity.

^a^Test for differences between boys and girls: Mann-Whitney *U* test for the differences in MEHHP, MEOHP, MnBP, and cotinine and *t*-test for outdoor environments; MEHHP, mono-(2-ethyl-5-hydroxyhexyl) phthalate; MEOHP, mono-(2-ethyl-5-oxohexyl) phthalate; MnBP, mono-n-butyl phthalate; PM_10_, particulate matter with a diameter less than 10 μm.

**Table 3:** Summary of respiratory outcomes of the study subjects.

Respiratory outcome	No	Total	Boys	Girls	*P*-value^a^
**PEFR (L/min)**	95	226.9 ± 54.1	228.8 ± 50.8	225.1 ± 57.1	0.755
**FEV_1_ (L)**	47	1.6 ± 0.4	1.6 ± 0.5	1.6 ± 0.4	0.730
**FEV_1_/FVC(%)**	47	84.2 ± 10.2	83.1 ± 11.2	85.5 ± 9.0	0.389
**FEF_25-75_ (%)**	47	69.4 ± 18.6	71.6 ± 19.7	67.4 ± 17.3	0.462
**FeNO (ppb)**	47	42.7 ± 23.7	41.3 ± 22.0	44.0 ± 22.7	0.688

Data are expressed as mean ± standard deviation

^a^ Test for differences between boys and girls: *t*-test for means of each variable; PEFR, peak expiratory flow rate; FEV_1_, forced expiratory volume in 1 second; FVC, forced vital capacity; FEF_25-75_, forced expiratory flow at 25% to 75% of FVC; FeNO, fractional exhaled nitric oxide.

The DIs of the phthalates were estimated using the Formula 1: DI of DEHP was estimated at 3.1 μg/kg body-weight(bw)/day (IQR, 2.2–5.4) and 3.8 μg/kg bw/day (IQR, 2.6–5.9) obtained from MEHHP and MEOHP, respectively. DI of DnBP based on MnBP was estimated at 7.6 μg/kg bw/day (IQR, 4.9–10.7).

Among 56 children, only 40 children measured their respiratory outcomes. A total of 47 pulmonary function data including FEV_1_, FEV_1_/FVC (%), FEF_25-75_ (%), and FeNO were collected from one measure of 22 children, two measures from 11, and three measures from 1 child. We also obtained 95 PEFR records from each of the 40 subjects; one measure of 7 children, two measures from 20, three measures from 4 children, and four measures from 9 children. The average values with standard deviations of respiratory outcomes are summarized in Table 3. There were no significant differences in the levels of pulmonary outcomes between boys and girls.

**Fig 1:**
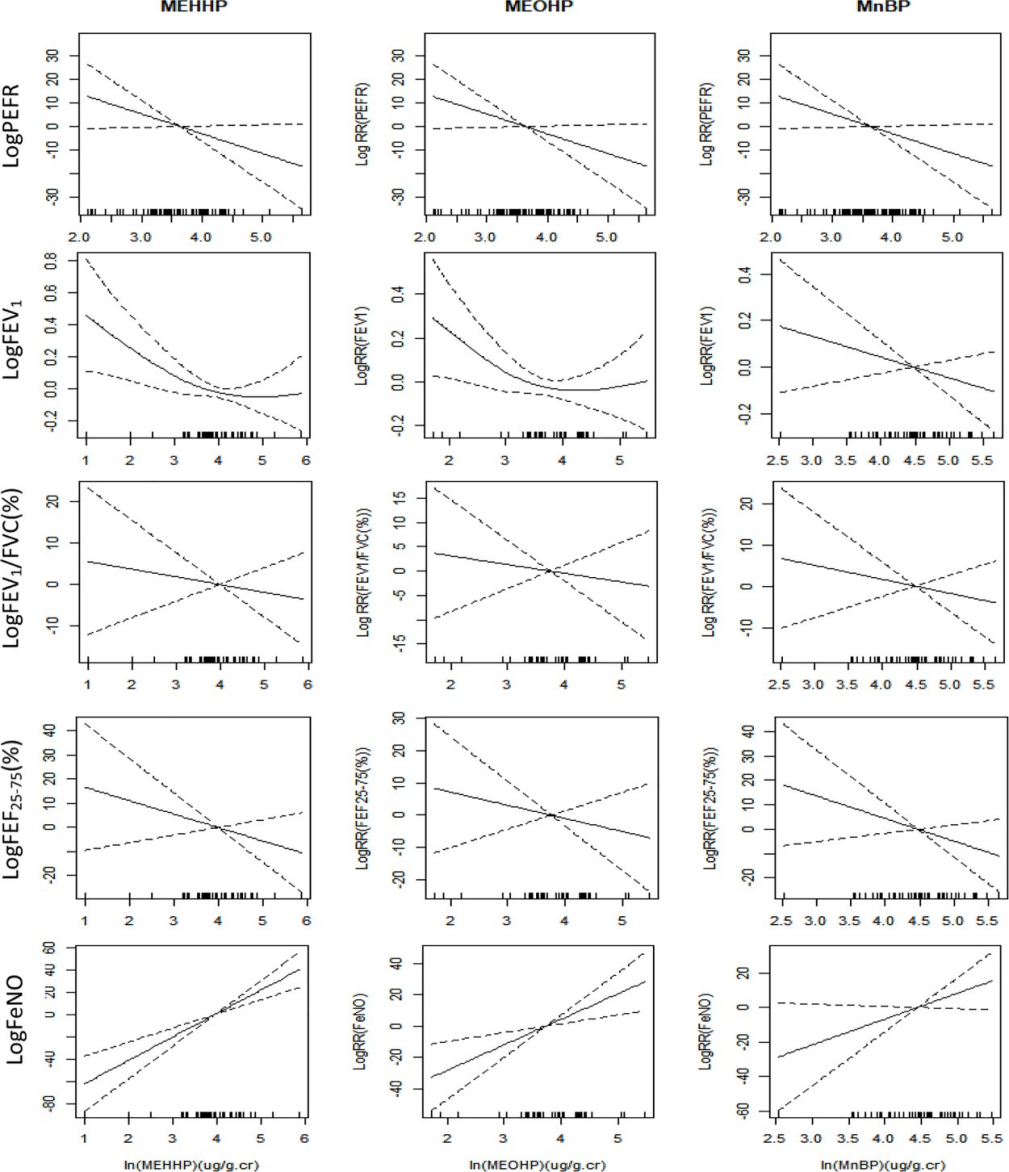
Relationship between urinary phthalate metabolites, pulmonary function and airway inflammation. FEV_1_, forced expiratory volume in 1 second; FVC, forced vital capacity; FEF_25-75_, forced expiratory flow at 25% to 75% of FVC; FeNO, fractional exhaled nitric oxide; MEHHP, mono-(2-ethyl-5-hydroxyhexyl) phthalate; MEOHP, mono-(2-ethyl-5-oxohexyl) phthalate; MnBP, mono-n-butyl phthalate; PEFR, peak expiratory flow rate.

Fig 1 demonstrates the relationships between ln-transformed levels of urinary phthalate metabolites and pulmonary functions or airway inflammation on the same day by the analysis of the GAMM, controlled for age, sex, BMI, urinary cotinine, the use of controller medication and outdoor environment. Pulmonary function (PEFR, FEV_1_, FEV_1_/FVC(%), and FEF_25-75_(%)) showed negative relationships with levels of urinary phthalate metabloites. FeNO showed positive linear relationships with all three urinary phthalate metabloites.

Table 4 shows the associations of phthalate exposures with respiratory outcomes for a ln-unit increase in urinary metabolites as a result of basic LME model adjusted for age, sex, BMI, urinary cotinine, and the use of controller medication and LME model with outdoor environmental indicators as confounders. As the urinary MEHHP, MEOHP, and MnBP levels increased, all the pulmonary function (PEFR, FEV_1_, FEV_1_/FVC(%), and FEF_25-75_(%)) tended to decrease in both basic LME model and the model with outdoor environmental indicators. However, only the association of MEHHP with FEV_1_ was statistically significant in that FEV_1_ decreased by 0.12 L [95% confidence interval (CI): 0.02, 0.22] as one ln-unit of MEHHP increased. Meanwhile, FeNO increased as the urinary phthalate metabolites increased. As one ln-unit of urine MEHHP and MEOHP increased, the FeNO level significantly increased by 13.77 ppb (95% CI: 4.71, 22.82) and 12.99 ppb (95% CI: 2.86, 23.12), respectively, according to the basic LME model. When ambient PM_10_, temperature, and RH were controlled, the FeNO level increased by 19.47 ppb (95% CI: 9.28, 29.67) and 17.93 ppb (95% CI: 5.86, 30.01), as MEHHP and MEOHP increased, respectively, showing slightly higher effect sizes compared to the basic model.

**Table 4:** Estimated regression coefficients for the relationship between urinary phthalates metabolites and respiratory outcomes on the same day.

Respiratory outcome	Phthalate metabolites	Estimate (95% confidence interval)	
		Basic model^a^	Model with outdoor environment^b^
**PEFR** **(L/min)**	MEHHP	-7.18 (-15.74, 1.39)	-8.40 (-17.39, 0.60)
	MEOHP	-7.28 (-16.25, 1.69)	-7.50 (-16.89, 1.90)
	MnBP	-5.28 (-13.31, 2.75)	-7.42 (-15.87, 1.04)
**FEV_1_ (L)**	MEHHP	-0.12 (-0.22, -0.02)*	-0.07 (-0.18, 0.03)
	MEOHP	-0.10 (-0.25, 0.04)	-0.04 (-0.16, 0.08)
	MnBP	-0.12 (-0.34, 0.09)	-0.13 (-0.29, 0.03)
**FEV_1_/FVC (%)**	MEHHP	-1.10 (-7.75, 5.55)	-1.91 (-8.63, 4.82)
	MEOHP	-0.10 (-7.31, 7.12)	-1.08 (-8.49, 6.34)
	MnBP	-2.21 (-12.41, 7.99)	-2.43 (-12.62, 7.77)
**FEF_25-75_ (%)**	MEHHP	-4.59 (-13.63, 4.46)	-4.27 (-14.01, 5.47)
	MEOHP	-2.42 (-12.31, 7.46)	-1.57 (-12.38, 9.24)
	MnBP	-8.17 (-22.05, 5.71)	-6.59 (-21.46, 8.28)
**FeNO (ppb)**	MEHHP	13.77 (4.71, 22.82)*	19.47 (9.28, 29.67)*
	MEOHP	12.99 (2.86, 23.12)*	17.93 (5.86, 30.01)*
	MnBP	10.40 (-5.03, 25.84)	16.42 (-2.09, 34.93)

All results were quantified according to natural log-transformed concentrations of urinary phthalate metabolites

*statistically significant

^a^results from basic LME model controlling for age, sex, body mass index, urinary cotinine, and the use of controller medication

^b^results from LME model with outdoor environments as additional confounders; MEHHP, mono-(2-ethyl-5-hydroxyhexyl) phthalate; MEOHP, mono-(2-ethyl- 5-oxohexyl) phthalate; MnBP, mono-n-butyl phthalate; PEFR, peak expiratory flow rate; FEV_1_, forced expiratory volume in 1 second; FVC, forced vital capacity; FEF_25-75_, forced expiratory flow at 25% to 75% of FVC; FeNO, fractional exhaled nitric oxide

Table 5 shows the lagged effects of phthalate exposure on PEFR in the LME model with adjustment for outdoor environment in addition to age, sex, BMI, urinary cotinine, and the use of controller medication. An increase in urinary MEHHP, MEOHP and MnBP was found to have significant lag effects on the PEFR; an increase in ln-transformed MEHHP, MEOHP and MnBP was related to a decrease in PEFR on the next day (LAG1) by 12.17 L/min (95% CI: 2.59, 21.74), 10.80 L/min (95% CI: 0.29, 21.32) and 13.65 L/min (95% CI: 5.07, 22.24), respectively.

**Table 5:** Estimated regression coefficients for the relationship between urinary phthalate metabolites and delayed responses of peak expiratory flow rate (PEFR).

Phthalate metabolites	Lagged day	Estimate (95% confidence interval)	
		Basic model^a^	Model with outdoor environment^b^
**MEHHP**	LAG0	-7.18 (-15.74, 1.39)	-8.40 (-17.39, 0.60)
	LAG1	-9.18 (-18.17, -0.18)*	-12.17 (-21.74, -2.59)*
	LAG2	-4.30 (-11.87, 3.28)	-5.00 (-12.70, 2.69)
**MEOHP**	LAG0	-7.28 (-16.25, 1.69)	-7.50 (-16.89, 1.90)
	LAG1	-9.42 (-19.39, 0.54)	-10.80 (-21.32, -0.29)*
	LAG2	-3.86 (-11.99, 4.26)	-2.74 (-11.04, 5.56)
**MnBP**	LAG0	-5.28 (-13.31, 2.75)	-7.42 (-15.87, 1.04)
	LAG1	-10.50 (-18.92, -2.07)*	-13.65 (-22.24, -5.07)*
	LAG2	-3.67 (-10.48, 3.13)	-5.47 (-12.23, 1.30)

All results were quantified according to natural log-transformed concentrations of urinary phthalate metabolites

*statistically significant

^a^results from basic LME model controlling for age, sex, body mass index, urinary cotinine, and the use of controller medication

^b^results from LME model with outdoor environments as additional confounders; all effects were quantified per natural log-transformed concentrations of urinary phthalate metabolites; MEHHP: mono-(2-ethyl-5-hydroxyhexyl) phthalate; MEOHP: mono-(2-ethyl- 5-oxohexyl) phthalate; and MnBP: mono-n-butyl phthalate.

## Discussion

In this longitudinal panel study, we found that increases in urinary phthalate metabolites were associated with decreased pulmonary function and increased airway inflammation in asthmatic children. Our results are consistent with previous studies [6,13,14], in which phthalate biomarkers were related to changes in respiratory outcomes although the subjective symptoms such as wheeze were used instead of objective outcomes such as FEV_1_. In addition, we found a significantly positive relationship between secondary metabolites of DEHP and FeNO, contrary to the previous study involving 244 children [14]. To the best of our knowledge, this is the first study reporting a significant relationship between DEHP exposure and FeNO in asthmatic children.

Interestingly, we also found that the adverse effect of phthalate exposure on pulmonary function may have time-lagged effect up to one day. In a human single oral dose study, about 71% of the DEHP dose was excreted after 24 h and an additional 4% of the dose was excreted in the next 20 h. In urine, the peak value of mono(2-ethylhexyl) phthalate (MEHP), the primary metabolite of DEHP, was observed only 2 h after administration, and MEHHP and MEOHP after approximately 4 h [22,26,27]. The short lag effect of DEHP and DnBP on PEFR may be related to the short physiological half-life of DEHP. However, more studies are required to elucidate the delayed effect of phthalate exposure on pulmonary function.

It is unclear how phthalates aggravate the pulmonary function and airway inflammation in asthmatic patients. One possible theory is that phthalate exposure induces oxidative stress and thus aggravates respiratory outcomes. Asthma exacerbation was associated with increased oxidative stress [28–30], and phthalates were related to increases in oxidative stress markers [31–35]. Studies from the National Health and Nutrition Examination Survey (NHANES) demonstrated that MnBP and mono-isobutyl phthalate (MiBP) were significantly associated with 8-hydroxydeoxyguanosine (OHdG), a biomarker of oxidative DNA damage. Additionally, the urinary concentrations of MBzP were associated with C-reactive protein, a nonspecific marker of systemic inflammation [31,34,35]. In a cross-sectional study of elementary school children in Korea, Kim et al. [32] demonstrated that the urinary concentrations of malondialdehyde (MDA), a product of lipid peroxidation, were significantly associated with the effect of several metabolites of phthalates (e.g., MiBP, MnBP, and MEHP). In a recent study analyzing the effect of phthalate on changes in pro-inflammatory cytokines at the gene, protein, and metabolite levels, repeated exposures to MEHP over several days led to elevated lipogenesis and lipid oxidation, suggesting that MEHP induces a pro-inflammatory state in differentiated adipocytes [33]. Therefore, phthalate exposure may increase pulmonary inflammation via induction of oxidative stress in asthmatic children. However, the comprehensive mechanism underlying the effect of phthalate exposures on respiratory health remains unclear.

Geometric mean values of urinary MEHHP, MEOHP, and MnBP in this study were 49.6 μg/g-creatinine (50th–95th percentile, 50.4–141.1), 38.4 μg/g-creatinine (50th–95th percentile, 37.3–102.5), and 71.8 μg/g-creatinine (50th–95th percentile, 69.9–206.6), respectively. These levels are comparable to those from a study of 1,030 non-asthmatic Korean children aged 3–18 years; geometric means of MEHHP, MEOHP, and MnBP were 49.7 μg/g-creatinine (50th–95th percentile, 48.0–223.3), 29.5 μg/g-creatinine (50th–95th percentile, 27.3–131.7), and 63.8 μg/g-creatinine (50th–95th percentile, 55.6–206.8), respectively [36], although MnBP level in our patients was slightly higher than in children in the general population. Of note, the levels of all three phthalate metabolites in Korean children are greater than those reported in 342 American children aged 6–11 years [37]. In addition, phthalate exposure level in young children was higher than that in old children in this study. Concentrations of phthalate metabolites under 8.6 years were greater than in those above 8.6 (*P*-values ≤ .0001 for all three metabolites), which suggested the need for additional investigation in infants exposed to phthalates.

In the present study, we estimated DIs for DEHP and DnBP. Based on the urinary MEHHP and MEOHP, the geometric means of DIs for DEHP were estimated at 3.1 μg/kg bw-day (range, 0.1–23.5) and 3.8 μg/kg bw-day (range, 0.6–27.6), respectively. The geometric mean of DI for DnBP was estimated at 7.6 μg/kg bw-day (range, 1.1–140.0) based on MnBP. Compared with RfD values of DEHP (20 μg/kg bw-day) and DnBP (100 μg/kg bw-day) proposed by USEPA [20], the geometric means of DIs for the asthmatic children in this study were lower. However, exposures to DEHP in two children and DnBP in one child were estimated to be greater than RfDs, suggesting that a few children, but not all, may require careful attention due to potential health risks associated with allergic and respiratory diseases.

Bekö et al. [38] reported that more than 90% of the total intake of DEHP was derived from sources other than indoor air and dust. A previous study also reported that consumption of dairy products or meat and the use of plastic packaging or storage were significantly associated with urinary DEHP metabolites [32], implying that oral intake is the main pathway of DEHP exposure. However, significant associations were also found between non-dietary exposures to DEHP in the indoor environment and allergic sensitization [13]. Therefore, all potential pathways of exposure to DEHP including ingestion and inhalation must be considered in asthmatic children to reduce the risk properly.

This study is limited because a small number of patients were enrolled from a single center. Neither FEV_1_/FVC (%) nor FEF_25-75_(%) showed statistical significance in the associations with phthalate exposure, and it might be due to the small sample size. Nevertheless, the association of phthalate exposure with FeNO and PEFR was robust and significant. Another possible limitation was that we used spot urine samples instead of 24-hour urine to determine the phthalate exposure in each patient. Urinary concentrations of phthalate metabolites were found to vary according to sampling time in a previous study [32], and most phthalates have short half-life [39,40]. It is still controversial whether the concentrations of phthalate metabolites in a single spot urine sample reflect the actual levels of daily exposure to phthalate. In contrast, a single measurement of urinary concentrations of phthalate metabolites in children represented a 6-month mean concentration, indicating a reasonable degree of temporal reliability [41]. Therefore, it might be appropriate to measure the phthalate metabolite levels in a single urine sample for epidemiologic study to evaluate the risk of allergic diseases [6]. It should be also considered that we did not control indoor environmental indicators such as PM_10_, volatile organic compound, temperature, and humidity in the statistical model, which is another weakness of our study.

### Conclusions

Our results suggest that exposures to specific phthalates such as DEHP and DnBP are significantly related to exacerbation of pulmonary function and airway inflammation in asthmatic children. Avoidance of exposure to phthalates may be needed to prevent aggravation of respiratory outcomes in asthma.

## Supporting information

S1 Dataset Data set for the analyses of the associations between phthalate exposures and respiratory outcomes in asthmatic children. (XLSX)

## Abbreviations

BBzP: butylbenzyl phthalate
BMI: body mass index
DnBP: di-n-butyl phthalate
DEHP: di-(2-ethylhexyl) phthalate
DEP: diethyl phthalate
FEF_25-75%_: forced expiratory flow at 25% to 75% of FVC
FeNO: fractional exhaled nitric oxide
FEV_1_: forced expiratory volume in 1 second
FVC: forced vital capacity
GAMM: generalized additive mixed model
IQR: interquartile range
LME: linear mixed effect model
MBzP: mono-benzyl phthalate
MEHHP: mono-(2-ethyl-5-hydroxyhexyl) phthalate
MEHP: mono(2-ethylhexyl) phthalate
MEOHP: mono-(2-ethyl-5-oxohexyl) phthalate
MnBP: mono-n-butyl phthalate
PEFR: peak expiratory flow rate
